# The incidence of cryptorchidism among boys in some provinces in Indonesia

**DOI:** 10.1186/1687-9856-2013-S1-P188

**Published:** 2013-10-03

**Authors:** Siska Mayasari Lubis, Vivekenanda Pateda, Aditya Suryansyah, I Made Arimbawa, Wayan Bikin Suryawan

**Affiliations:** 1 Pediatric Endocrinology Division, Department of Child Health, Medical School, University of Sumatera Utara, Medan, North Sumatra, Indonesia; 2 Pediatric Endocrinology Division, Department of Child Health, Medical School, University of Sam Ratulangi, Prof.RD Kandou Hospital, Manado, North Sulawesi, Indonesia; 3 Pediatric Endocrinology Department, Harapan Kita Hospital, Jakarta, Indonesia; 4 Pediatric Endocrinology Division, Department of Child Health, Medical School, Udayana University, Sanglah Hospital, Bali, Indonesia

## Background

Cryptorchidism is a condition in which one or both testes are not fully descended to the bottom of the scrotum. It may be an important cause for male infertility. Numerous epidemiological studies indicate that the incidence has increased in many countries. The data about its incidence in Indonesia is still incomplete.

## Aims

The aim of this study was to establish the incidence of cryptorchidism in some provinces in Indonesia.

## Methods

This was a retrospective, multicentre descriptive study, we collected data from hospital based registry data that reported by pediatric endocrinologists from North Sumatera, North Sulawesi, Jakarta, and Bali provinces, Indonesia, from 2006 till 2012.

## Results

From the registry data there are 274 patients that were diagnosed with cryptorchidism. It was 29.56% for boys under 6 months, 31.39% for those between 6 months and one year, and increased significantly in older boys (39.05%). Overall 43.07% were diagnosed with bilateral cryptorchidism, 29.56% with left unilateral and 27.37% with right unilateral cryptorchidism.

## Conclusion

Our data showed a relatively higher prevalence of cryptorchidism in children older than 2 years of age, which may be caused by late diagnosis. We need to increase the awareness of this condition among public population and medical providers.

